# Distribution, Multi-Index Assessment, and Sources of Heavy Metals in Surface Sediments of Zhelin Bay, a Typical Mariculture Area in Southern China

**DOI:** 10.3390/toxics11020150

**Published:** 2023-02-03

**Authors:** Yan-Jie Han, Rui-Ze Liang, Hai-Song Li, Yang-Guang Gu, Shi-Jun Jiang, Xiang-Tian Man

**Affiliations:** 1 College of Marine Ecology and Environment, Shanghai Ocean University, Shanghai 201306, China; 2 South China Sea Fisheries Research Institute, Chinese Academy of Fishery Sciences, Guangzhou 510300, China; 3 School of Environment, Jinan University, Guangzhou 510632, China; 4 College of Life Science and Technology, Jinan University, Guangzhou 510632, China; 5 Southern Marine Science and Engineering Guangdong Laboratory (Zhuhai), Zhuhai 519000, China; 6 College of Oceanography, Hohai University, Nanjing 245700, China; 7 Institute for Environmental and Climate Research, Jinan University, Guangzhou 510632, China

**Keywords:** heavy metals, sediments, risk assessment, contamination, Zhelin Bay

## Abstract

The occurrence, multi-index assessment, and sources of heavy metals in surface sediments of Zhelin Bay were investigated. Average heavy metal concentrations (mg/kg) were 81.89 (Cr), 770.76 (Mn), 16.81 (Co), 62.25 (Ni), 96.30 (Cu), 162.04 (Zn), and 73.40 (Pb), with the concentrations of studied seven heavy metals being significantly higher than their corresponding background values. Geo-accumulation index (*I_geo_*) and pollution load index (PLI) were implemented to assess degree of heavy metal contamination. The *I_geo_* and PLI indicated that Cr, Mn, Co, Zn, and Pb were slightly polluted, and Cu and Ni were moderately polluted in the region. Potential ecological risk index (RI) and mean possible effect level (PEL) quotient were conducted to assess ecological risk. The RI and mean PEL quotient demonstrated that surface sediments of Zhelin Bay were slight ecological risks and exhibited a 21% probability of toxicity. Principal component analysis (PCA) combined with the correlation analysis (CA) and hierarchical cluster analysis (HAC) revealed that the heavy metal contamination in Zhelin Bay might originate from three type sources.

## 1. Introduction

The coastal zone is the land–sea border, with a favorable geographic location and abundant natural resources that not only provide us with sufficient food, but also create a good environment for human activity [1,2,3]. The biological environment in coastal areas is under considerable stress due to rapid economic development and massive anthropogenic activities [4,5,6]. Heavy metal pollution is one of the most crucial environmental issues in aquatic ecosystems [7,8,9] because heavy metals are persistent and highly toxic, and they tend to accumulate in organisms [4,10,11]. When heavy metals enter the coastal ecosystems, a major part of them accumulates in sediments through precipitation, adsorption, and diffusion processes [4,12,13]; however, this process can be reversed when the equilibrium between sediments and the overlying water body is broken, making sediments successively release heavy metals into water body, which may adversely affect the aquatic ecosystems and harm human health via the food chain [4,5,12].

Over the past 20 years, China has contributed more than 56% of the world’s aquaculture products [4,14]. In addition, China is also the world’s largest consumer and exporter of fishery products [4,15]. The fact that the quality of Chinese aquatic products is directly affected by the production environment has a considerable impact on consumer safety worldwide and needs to be fully investigated. Guangdong is an economically developed province and also the largest aquaculture area in China. Zhelin Bay, a semi-enclosed bay with its mouth facing south, is located in northeastern Guangdong Province, and it is one of the bays most significantly affected by mariculture in China [4,16]. In recent years, many studies have been conducted on heavy metal pollution in Zhelin Bay [16,17,18]. Various methods, such as geo-accumulation index method, enrichment factor method, pollution load index method, sediment quality guideline method, and potential ecological risk evaluation were implemented to evaluate the degree of heavy metal pollution in the surface sediments of Zhelin Bay. However, whether the results of the evaluation using these methods are uniform and whether they provide an objective assessment of the pollution level needs to be further studied. Therefore, the objectives of this study were to (1) investigate the heavy metal concentration and distribution characteristics in surface sediments of Zhelin Bay, (2) comprehensively assess the risk of heavy metals, and (3) identify potential sources.

## 2. Materials and Methods

### 2.1. Study Area and Sampling

**Figure 1:**
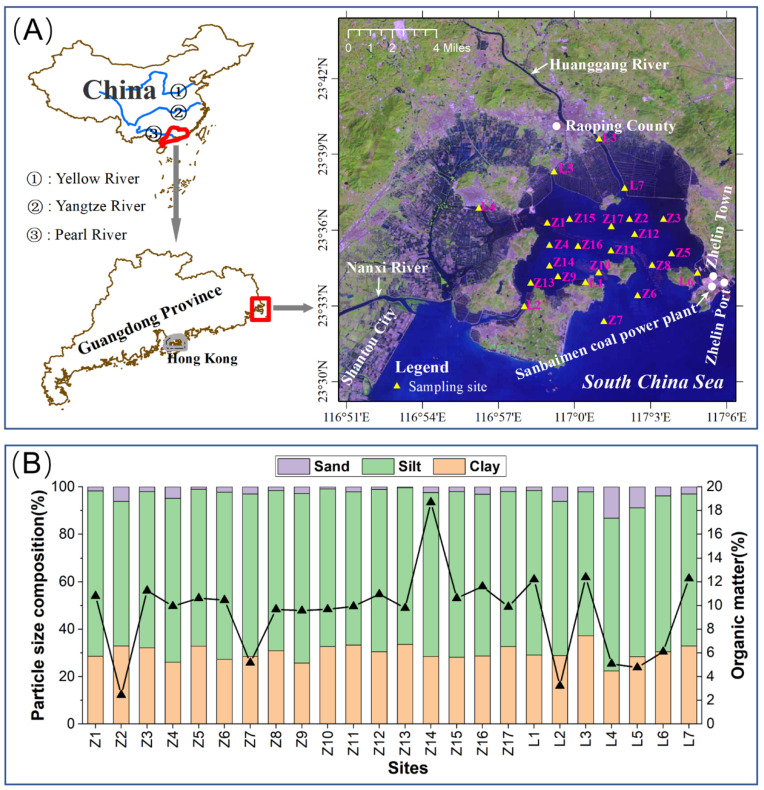
Map of the study area and sampling sites of Zhelin Bay, South China (**A**), as well as spatial variations of grain size compositions and organic matter in surface sediments of Zhelin Bay (**B**).

Zhelin Bay covers an area of ~70 km^2^ and is surrounded by land on three sides [4]. Huanggang River flows into the bay and is the only source of surface runoff input to Zhelin Bay (Figure 1A). The aquaculture industry is progressively developed in Zhelin Bay, which is the largest demonstration base of seawater net-box culture in China and one of the largest seawater culture areas in Guangdong Province. The eastern and western parts of the bay are shellfish culture areas, and the central part is cage culture area by our investigation. The uniform distribution strategy was adopted for the design of stations and the impact of rivers/discharge channels on Zhelin Bay was also considered. The sediments of Zhelin Bay are mainly composed of clay minerals [19]. In September 2021, Peterson grab sampler was used to collect surface sediments from twenty-four sites (Figure 1A). The surface layer (0–5 cm) sediment samples are encapsulated in sealed bags, numbered, recorded, and stored at −20 ℃ until brought back to the laboratory for analysis.

### 2.2. Sample Processing and Chemical Analysis

The samples were dried to constant weight in a constant temperature drying oven at 60 °C, and large pieces of calcareous debris and rocks were removed. The samples were ground using an agate mortar and passed through a 200 mesh (<74 μm) plastic sieve for determining heavy metals and the mass fraction of organic matter in the sediments.

X-ray fluorescence spectrometry (XRF) (Skyray, Shenzhen, China) was used to determine metal content. Pressed powder tablets were prepared from ground sediment samples (<74 μm), followed by direct elemental determination of the samples [16]. The China National Standard material (Offshore Marine sediment, GBW 07314) was conducted to verify the XRF recovery. Recoveries for the seven metals were between 98% and 110%.

The organic matter (OM) content of the sediment was measured through loss on ignition (LOI) [20]. Namely, dry sediment was incinerated at 550 °C for 4 h to estimate OM content. The particle size was determined by Mastersizer 3000 laser diffraction particle size analyzer. About 2 g wet sediment sample was placed in a 50 mL centrifuge tube and placed in a constant temperature water bath. An amount of 30% H_2_O_2_ and 15% HCl were added to remove organic matter and carbonates, and 10 mL 0.1 mol/L sodium hexametaphosphate (NaPO_3_)_6_ was added to ensure adequate dispersion of the sample prior to analysis.

### 2.3. Statistical Analysis

The mean concentrations of Cr, Mn, Co, Ni, Cu, Zn, and Pb in the sediments were compared to their corresponding background values using a one-sample t-test of the SPSS v26.0 (IBM Corp, Armonk, NY, USA) for Windows. The data of Cr, Co, Ni, Cu, Zn, Al, Fe, and TOC were Box-Cox transformed using STATISTICA for Windows v8.0, and the transformed data conformed to the normal distribution. The transformed data were then standardized. The correlation analysis (CA), hierarchical clustering analysis (HCA), and principal component analysis (PCA) were performed based on data standardized. PCA was also used in SPSS v26.0 for Windows. Pearson correlation analysis and hierarchical clustering analysis were performed using R v3.6.2 (The University of Auckland, Auckland, New Zealand). Inverse distance weighted (IDW) analysis was used to elucidate the spatial distribution characteristics of metal elements. Sampling station maps and IDW interpolation values were generated by ArcGIS 10.2 (Environmental Systems Research Institute, Inc., RedLands, CA, USA).

## 3. Results and Discussion

### 3.1. General Characteristics of Sediments

The general characteristics of the sediment were obtained by measuring particle size composition and organic matter. The organic matter contents ranged from 2.44% to 18.70% of dry weight, with an average of 9.47%. The grain size of sediment can better reflect the source, transport mode, hydrodynamic condition, and so on. It is a carrier for recording information on sediment transfer and transport. The particle size composition of sediments has an influence on the adsorption and transport of heavy metals [21]. The sediments in the survey area consist mainly of silt, followed by sand and clay (Figure 1B). The most widely distributed sediment type in the inner bay of Zhelin Bay is clay and silt clay, and the nearshore area is dominated by medium and coarse sand, and L7 in the estuary area, Z2, and L6 near Xi’ao Island belong to this sediment type [22].

### 3.2. Concentrations and Distribution of Heavy Metals

The concentration ranges of studied heavy metals in surface sediments were: 43.40–98.88 mg/kg for Cr, 433.60–1132.90 mg/kg for Mn, 10.00–19.90 mg/kg for Co, 46.90–123.10 mg/ kg for Ni, 81.13–159.33 mg/kg for Cu, 138.90–201.70 mg/kg for Zn, and 53.70–85.90 mg/kg for Pb (Table 1). The average concentrations of heavy metals were Mn > Zn > Cu > Cr > Pb > Ni > Co. The results of the one-sample t-test showed that the concentrations of Cr, Mn, Co, Ni, Cu, Zn, and Pb in the sediments of Zhelin Bay were significantly higher than their corresponding background values. The relatively high metal concentrations in Zhelin Bay may be due to anthropogenic activities such as surface runoff, sewage discharge, and mariculture (Figure 1A). 

Compared the content of heavy metals in Zhelin Bay with other bays [23,24,25,26,27,28,29,30], the average concentrations of studied heavy metals in Zhelin Bay were higher than those in Quanzhou Bay, Shantou Bay, Daya Bay, Pearl River Estuary, Qinzhou Bay, and San Francisco Bay in the United States (Table 1). In order to protect and manage China’s coast, the Chinese government issued the Chinese Marine Sediment Quality (GB18668-2002) standard [31]. According to the standard, marine sediment quality is divided into three categories: Class I applies to marine nature reserves, mariculture areas, seawater bathing areas, and human sports and recreation; Class II can be used for industrial water areas and tourist areas; marine port waters and marine development operation areas use Class III standards. Compared the average concentrations of heavy metals in the surface sediment of Zhelin Bay with the quality standard, it was found that, except for Cr at seven sites, Zn at two sites, and Pb at three sites, which were below the threshold of Class I quality, all the others belonged to Class II, and Cu at all sites belonged to Class II quality, indicating that the heavy metal pollution in the sediment posed ecological risks to marine organisms.

**Figure 2:**
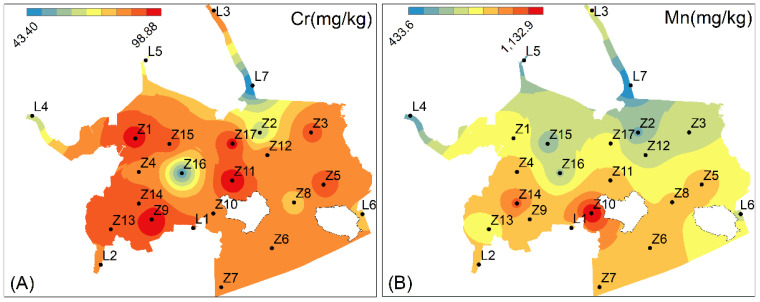

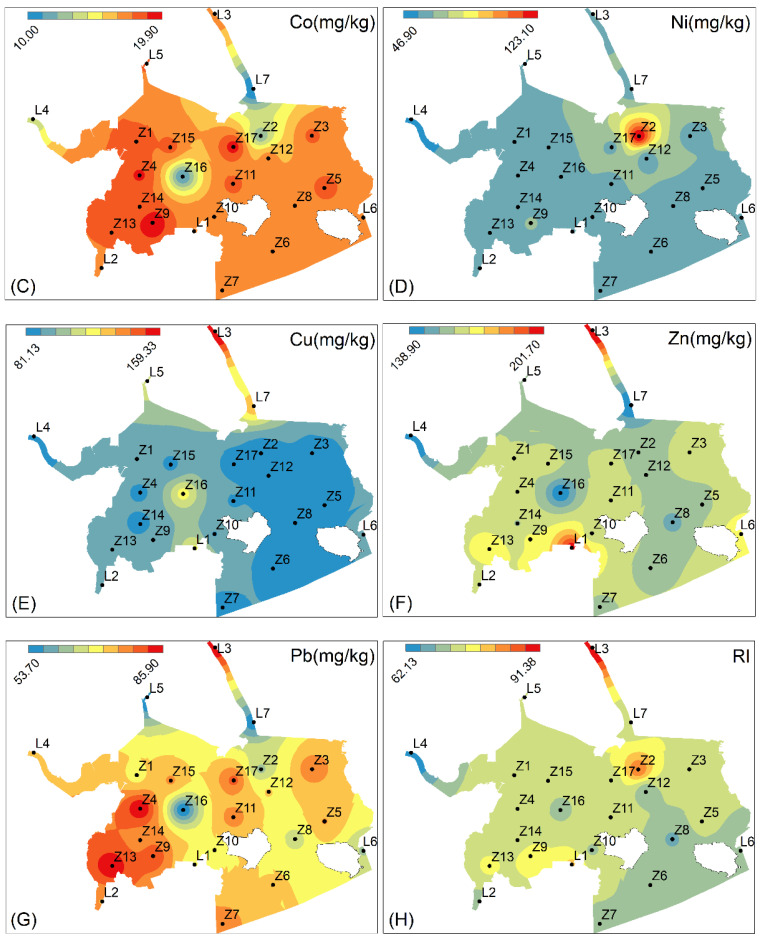
Spatial distribution of Cr, Mn, Co, Ni, Cu, Zn, and Pb (**A**–**G**) and potential ecological risk index (RI) in surface sediments of the Zhelin Bay (**H**).

Moreover, the spatial distributions of heavy metals in the study area were described by IDW interpolation analysis (Figure 2). The spatial distributions of Cr, Mn, Co, Zn, and Pb are generally consistent, with lower levels at stations L7 and Z2 near the mouth of the Huanggang River, station Z16 near the navigation channel, and station L6 in the narrow waterway near Xi’ao Island, as well as higher levels in the area surrounded by the North and South Haishan Islands. The higher Ni content at Z2 may be due to the large number of fishing vessels that frequently sail and moor in the vicinity. In addition, the highest values of Cu, Zn, and Pb occur in the area through which the Huanggang River flows. Surface runoff from the water bodies of Huanggang River may be the source of Cu, Zn, and Pb pollution.

### 3.3. Multi-Index Assessment

#### 3.3.1. Geo-Accumulation Index (*I_geo_*)

The geo-accumulation index (*I_geo_*) is a method to evaluate the degree of heavy metal pollution in sediments by using the relationship between the total amount of heavy metals and their background values [35]. The calculation formula is as follows [36]:

$$I_{geo}{=\log}_{2}\left(\frac{C_{n}}{1{.5B}_{n}}\right)$$

where C_n_ is the measured value of heavy metal element (n) in the sample, B_n_ is the background value or is the crustal shale value of metal element (n), and factor 1.5 is the correction index, which is chosen after considering the variation in background values that may be caused by rock differences in different places. The sediment can be divided into seven classes by the geo-accumulation index (*I_geo_*) (Table S1). 

**Figure 3:**
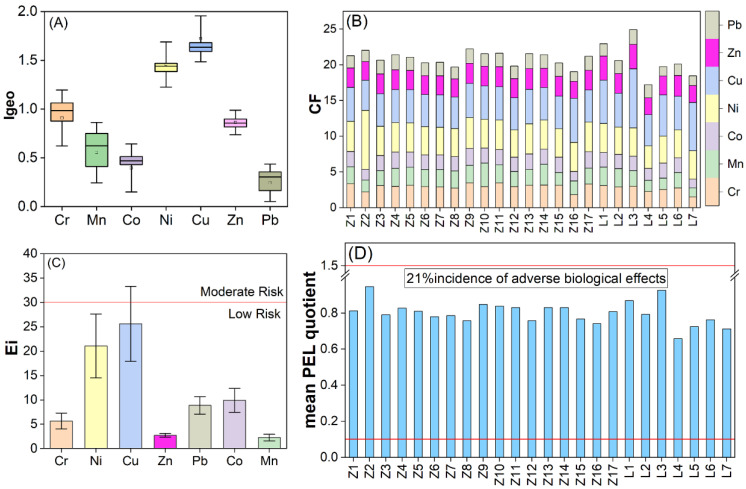
Contamination assessment results of Zhelin Bay: *I_geo_* value (**A**), contamination factor (CF) (**B**), single element ecological risk factor ($E_{r}^{i}$) (**C**), and mean PEL quotient (**D**).

The ranges of *I_geo_* values were: 0.02–1.20 for Cr, −0.25–1.14 for Mn, −0.34–0.66 for Co, 1.07–2.46 for Ni, 1.49–2.47 for Cu, 0.65–1.18 for Zn, and −0.18–0.50 for Pb. The average values of *I_geo_* for heavy metals in Zhelin Bay were Cu (1.72) > Ni (1.46) > Cr (0.91) > Zn (0.86) > Mn (0.55) > Co (0.40) > Pb (0.24). The *I_geo_* values of Pb and Co at all stations were less than 1, indicating that these two elements were in the state of uncontaminated to moderate pollution; the *I_geo_* values of Zn, Mn, and Cr at some stations were between 1 and 2, while the average *I_geo_* values at all stations were less than 1, indicating that the area is slightly polluted by these three elements. In addition, the *I_geo_* values of Cu and Ni in Zhelin Bay were between 1 and 2, indicating moderate pollution by these two elements, and the *I_geo_* values of some stations exceeded 2 (e.g., the *I_geo_* value of Ni at site Z2 reached 2.46, and the *I_geo_* value of Cu at L3 was 2.47), which belonged to the state of moderate to strong pollution (Figure 3A).

#### 3.3.2. Pollution Load Index (PLI)

The contamination factor (CF) was used to describe the level of contamination of a single metal in the sediments. The calculation equation was as follows [37]:

$$\mathrm{CF}=\frac{C_{\mathrm{heavy}\;\mathrm{metal}}}{C_{\mathrm{background}}}$$

where C_heavy metal_ is concentration of studied metal in sediments; C_background_ is background concentration of heavy metal. CF < 1 indicates very low contamination, 1 ≤ CF < 3 denotes moderate contamination, 3 ≤ CF < 6 implies severe contamination, and CF ≥ 6 represents very high contamination [37]. According to the calculations, the average CF of Cr, Mn, Co, Zn, and Pb ranged from 1 to 3 and were moderately contaminated, while the average CF of Ni and Cu ranged from 3 to 6, indicating severe contamination of Ni and Cu in the sediment. The average CF of each metal in the sediment was Cu > Ni > Cr > Zn > Mn > Co > Pb (Figure 3B).

Since heavy metals are not present singularly in sediments, the pollution load index (PLI) proposed by Tomlinson et al. [38] was used to describe the combined pollution at each sampling site in the study area. It was calculated as follows [18]:

PLI=CF1×CF2×CF3×⋯×CFn1n

where *n* is the number of heavy metals (here, seven) and CF_n_ is a contamination factor. PLI values were classified into two levels: uncontaminated (PLI < 1) and contaminated (PLI > 1). [18] further classified PLI as follows: (1 < PLI ≤ 2) represents light pollution; (2 < PLI ≤ 3) represents moderate pollution; (3 < PLI ≤ 4) represents moderate to severe pollution; (4 < PLI ≤ 5) represents severe pollution; and (PLI > 5) represents very severe pollution [18]. The PLI of each sampling site ranged from 2.13 to 3.14. The PLI of three sites, Z9, L1, and L3, were greater than 3, which were moderately to severely polluted. The PLI at the other stations ranged from 2 to 3, indicating moderate contamination of the investigated surface sediments of Zhelin Bay (Figure S1).

#### 3.3.3. Potential Ecological Risk

Potential ecological risk index (RI) was a method proposed by Swedish scientist Håkanson [37] to evaluate heavy metal contamination in sediments or soils from a sedimentological point of view, based on the nature of heavy metals and their environmental behavior. This method links heavy metal content, ecological effects, environmental effects, and toxicology, and it is suitable for evaluation and comparison between different sediments on a large regional scale. According to Håkanson’s method [37], the single element pollution index ($C_{f}^{i}$), single element ecological risk factor ($E_{r}^{i}$), and multi-element potential ecological risk index (RI) of heavy metal i in sediments of a certain area can be calculated by the following equations:

$$C_{f}^{i}=\frac{C^{i}}{C_{0}^{i}}$$

$$RI=\underset{i=1}{\sum }E_{r}^{i}=T_{r}^{i}\times C_{f}^{i}$$

where $C_{f}^{i}\;$ is the pollution coefficient of heavy metal i; $C^{i}$ is the sample measurement; $C_{0}^{i}\;$ is the environmental background value; and $T_{r}^{i}\;$ is the heavy metal toxicity response coefficient (Cr = 2, Cu = Co = Ni = Pb = 5, Mn = Zn = 1). $E_{r}^{i}\;$ and RI pollution intensity grades are shown in Table S2.

The $E_{r}^{i}$ results showed that Cr ranged from 3.03 to 6.91; Mn was from 1.26 to 3.30; Ni varied from 15.74 to 41.30; Cu was from 21.10 to 41.75; Zn ranged from 2.34 to 3.40; Pb varied from 6.64 to 10.63; and Co was from 5.95 to 11.83. Cu reached a maximum value of 41.75 at L3 and showed moderate hazard at L1, L3, and L7. Ni was 41.30 at Z2, reaching a moderate hazard level, and slight hazard existed at the rest of the stations. Additionally, $E_{r}^{i}$ of four metals, Co, Pb, Cr, and Zn, were at a low level (Figure 3C). The mean $E_{r}^{i}$ order was: Cu (25.08) > Ni (20.89) > Co (10.00) > Pb (8.96) > Cr (5.73) > Zn (2.73) > Mn (2.24). Consistent with the results of *I_geo_* and CF, Cu mainly contributed to RI. The contribution of $E_{r}^{i}$ (Cu) to RI ranged from 25% to 47%, with an average of 34%, indicating that Cu is the main potential ecological risk in Zhelin Bay. The spatial distribution of RI ranged from 63.71 to 93.55, which was in the slight class (RI < 100) (Figure 2H). The maximum value is located at sampling site L3, which has surface runoff input from Huanggang River and is influenced by human activities. 

#### 3.3.4. Sediment Quality Guidelines (SQGs)

Sediment quality guidelines (SQGs) are effective guidelines for assessing pollutant ecological risk in marine and estuarine sediment [39]. The main parameters for estimating the adverse biological effects of metals, namely, threshold effect levels (TELs) and probable effect levels (PELs), are presented in the SQGs. Adverse effects rarely occur at metal concentrations below the TELs; they frequently occur at metal concentrations above the PELs [34]. The TEL and PEL values of Cr, Mn, Ni, Cu, Zn, and Pb are shown in Table 1. Accordingly, the results showed that the concentrations of Cu at 21% of the stations and Ni at all stations were greater than the corresponding PELs, indicating that Cu and Ni at these stations tend to exhibit adverse biological effects; 92% of Cr, 96% of Mn, and Zn and Pb concentrations at all stations were between TEL and PEL, indicating that Cr, Mn, Zn, and Pb in most samples occasionally show potential ecological risks (Table 1).

Heavy metals often occur as complex mixtures in sediments. We determined the biological effects of mixed toxic groups by calculating the mean possible effect level (PEL) quotient. The formula was as follows [40]:

$$\mathrm{mean}\;\mathrm{PEL}\;\mathrm{quotient}=\sum \left(C_{x}/PEL_{x}\right)/n$$

where *C_x_* is the measured concentration of metal *x*, *PEL_x_* is the PEL for metal *x*, and *n* is the number of metals. The classification is based on the probability of acute toxicity in marine sediments, with the incidence of adverse biological effects being 8%, 21%, 49%, and 73% when the mean PEL quotient is below 0.1, 0.1 to 1.5, 1.5 to 2.3, and above 2.3, respectively [41]. As shown in Figure 3D, the mean PEL quotient of the surface sediments in Zhelin Bay varied in the range of 0.66–0.94, indicating a 21% incidence of adverse biological effects for the studied metal combinations.

#### 3.3.5. The Relationships among Multiple Indexes

**Figure 4:**
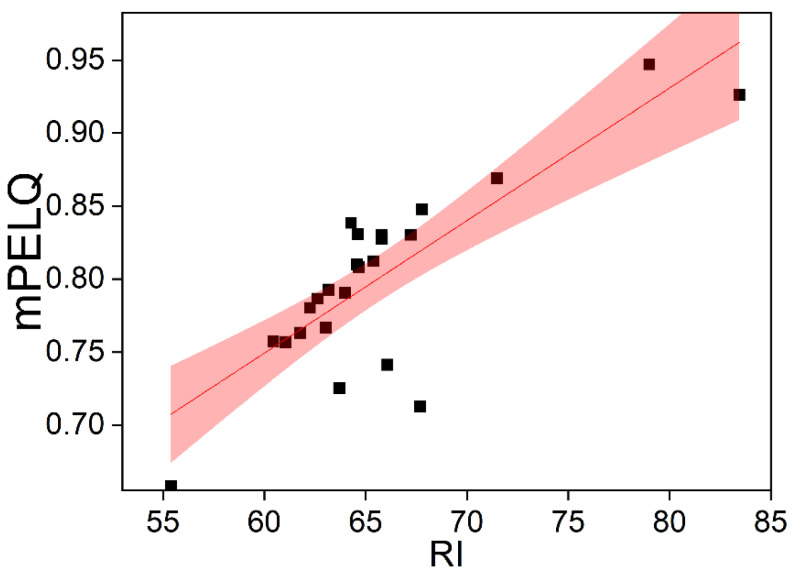
Correlation between the potential ecological risk index (RI) and mean PEL quotient for heavy metals in surface sediments of different stations in Zhelin Bay (R^2^ = 0.81, *p <* 0.05).

In this study, the results of the evaluation of the surface sediments of Zhelin Bay using the *I_geo_* and the CF were consistent (See Section 3.3.1 and Section 3.3.2). The SQGs and RI link pollutants to their biotoxicity, take into account the toxicity differences of different heavy metals, and reflect the combined effects of multiple pollutants. Figure 4 shows the correlation between the potential ecological risk index of each station and the mean PEL quotient (R^2^ = 0.81, *p* < 0.05). The contamination degree and change trend of the stations reflected by the potential ecological risk index (RI) and the mean PEL quotient are basically consistent, revealing that there is a slight ecological risk of heavy metals in the surface sediments of Zhelin Bay.

### 3.4. Source Analysis of Heavy Metals

**Figure 5:**
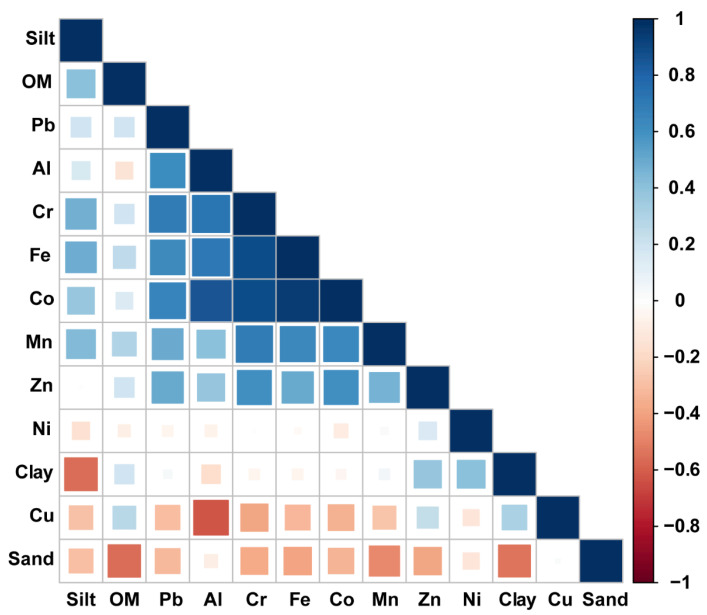
Pearson correlation coefficients (r) among the heavy metals along with organic matter and grain sizes. Data in the boxes show significant correlations (*p* < 0.05) between the elements.

The correlation analysis between heavy metal elements and characteristic parameters in sediments allows the sources of heavy metals and their content variation control factors to be determined [42]. The elements are significantly correlated when the sources in the sediments are the same or similar [43]. If there is no correlation between elements, metals are not controlled by a single factor [44]. To explore the intrinsic connection between heavy metal pollution and environmental factors in Zhelin Bay, Pearson correlation analysis was performed between heavy metal elements and sediment particle size composition, and the results are shown in Figure 5. Fe and Cr, Al, Pb, Co, Mn, and Zn were significantly correlated (*p* < 0.05) (Table S3), indicating that these elements mainly originated from rock sources. Cu was negatively correlated with the concentrations of the elements, with the exception of Al, which had no significant correlation with the other elements, indicating that it may not be of the same origin as the other metals. There is no significant correlation between Ni and other metallic elements, indicating that its origin is not controlled by a single factor. As shown in Figure 5, all heavy metals are negatively correlated with sand; except Cu and Ni, the rest of heavy metals are positively correlated with silt, indicating that the fine-grained level is conducive to heavy metal enrichment [45].

Overall, the spatial content distribution of Cr, Co, Zn, Pb, Fe, and Mn is roughly the same, and the metal element content in the bay shows a higher content in the west than in the east. At stations L7 and Z2 at the mouth of Huanggang River and station L6 at the narrow waterway in the southeast of Zhelin Bay and station Z16 near the channel, it is difficult to deposit fine particulate matter due to the influence of hydrodynamics, therefore, the heavy metal contents at these four stations are obviously lower than those at other stations. In the inner bay of Zhelin Bay, due to the blockage of oyster piles, fish rafts and nets set by human, the water flow is slow, water exchange is weakened, fine particulate matter is deposited, and the heavy metal content is relatively high. Previous studies have shown that the main causes of high heavy metal concentrations in Zhelin Bay are sewage discharge, land-based input, port activities, and mariculture [16,46]. In addition, as a port and energy and petrochemical base in Guangdong Province, the pollution sources of gasoline and diesel combustion in Zhelin Bay cannot be ignored [47].

**Figure 6:**
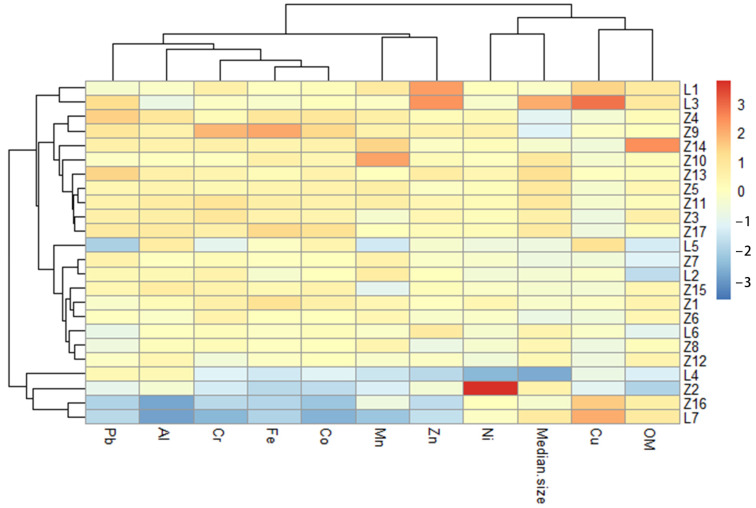
Dual hierarchical clustering analysis for sampling stations and heavy metals.

The dual-level clustering analysis of heavy metals and sampling stations is shown in Figure 6. The horizontal dendrogram shows the clustering of nine metal concentrations as well as OM and median particle size in 24 samples. The results can be divided into three clusters: Cr, Co, Zn, Pb, Fe, Mn, and Al (cluster 1), Ni and median particle size (cluster 2), and Cu and OM (cluster 3). The clustering analysis results were consistent with Pearson correlation analysis. Vertical dendrograms show the clustering of all sampling stations in the 24 samples. An amount of 24 sampling sites yielded two main distinct sample groups. One group is mainly located in estuaries and waterways; the other group mainly includes mariculture areas. The results of the cluster analysis also indicated that heavy metal pollution in the sediments of Zhelin Bay is influenced by mariculture and other human activities.

Principal component analysis (PCA) was used to determine the potential sources of heavy metal elements in the sediments (Table 2). The PCA results identified three principal components (PC1-3), which explained 76.85% of the total variance. The variance of PC1 was 46.78%, in which Co, Cr, Fe, Al, Pb, Mn, and Zn had high positive loadings, indicating that they mainly originated from the weathering of the rocks. The soil type in the east of Guangdong is ruddy loam, rich in Fe and Al, which may explain the high Fe and Al content [48]. However, combining the *I_geo_* index with the pollution index of individual elements, the elements of Cr, Co, Zn, and Pb not only originate from nature, but are also influenced by human factors. The high correlation of Cr, Co, Zn, and Pb with Fe and Mn indicates that oxides or hydroxides of Fe or Mn are the main fugitive phases of these metals, which are mainly present in the sediment as a combination of Fe oxides and Mn oxides and migrate from the water column to the sediment through adsorption [49]. Cr, Mn, Co, Zn, and Pb are high in the semi-enclosed bay area of the northwest coast and near the port of Sanmen, probably because the port is mainly engaged in trade and fishing. Environmental problems such as urban pollution, port construction, and shipping waste caused the high content of heavy metals. Meanwhile, four elements, Cr, Mn, Co, Zn, and Pb, were high in heavy metals within the southern and northern part of Haishan Island, an area that is a shellfish farming area and mainly influenced by mariculture [50]. The feeds put in mariculture contain heavy metals [51,52]. Mariculture puts a large amount of feed to increase production, but the vast majority of it cannot be used by farmed fish, thus making the sediment contain a large amount of organic matter, which in large amounts combines with heavy metals and further enhances the enrichment of heavy metals [18,53]. 

The second principal component (PC2, accounting for 21.41% of the total variance) has larger loadings for Cu and OM, as well as moderate loadings for median grain size and Zn. Cu is negatively correlated with each element and positively correlated with OM, indicating that Cu pollution is correlated with OM and grain size. Cu and Zn may have the same source. The maximum values of Cu and Zn occur at station L3, through which the Huanggang River flows, indicating that the pollution mainly comes from land-based runoff input. The maximum values of Cu and Zn occur at station L3, through which the Huanggang River flows, indicating that the pollution mainly comes from land-based runoff input. On the one hand, the eastern part of Guangdong Province is one of the six major mining areas with abundant copper ore resources, and the products of ore erosion and weathering enter the bay through surface runoff under the action of water or wind; on the other hand, human production and domestic wastewater and industrial and agricultural discharge also enter Zhelin Bay through the Huanggang River. The pollution of Cu and Zn in the inner bay of Zhelin Bay mainly comes from sewage and the feed put in the mariculture activities [12]. Cu and Zn are important elements in commercial fish feeds and are growth promoters for fish [50]. In addition, antifouling coatings are widely used in aquaculture nets. A large amount of metal elements dissolve from antifouling coatings and enter the sediment.

The third principal component (PC3, 11.61%) exhibited strong positive loadings of Ni and median grain size. Ni has little spatial variation in the inner bay of Zhelin Bay, prominent only at station Z2. Ni is widely used as a plating for ship protection because of its resistance to seawater corrosion, and it is also a trace metal element in gasoline [12,47]. In the netted mariculture area, fishing vessels are active, and the burning of gasoline and diesel by vessels is the main source of pollution from Ni.

## 4. Conclusions

Heavy metal concentrations (Cr, Mn, Co, Ni, Cu, Zn, and Pb) in surface sediments from 24 sites of Zhelin Bay exhibited significant spatial variability. Concentrations of studied heavy metals significantly exceeded their background values, indicating anthropogenic enrichment. Multi-index assessment was applied to assess the degree of heavy metal contamination and ecological risk, which helped us to more comprehensively understand the degree of heavy metal contamination and potential ecological risk of heavy metals. The *I_geo_* and PLI revealed that most studied heavy metals had slight contamination. The RI coupled with mean PEL quotient indicated that surface sediments of Zhelin Bay had slight ecological risk and exhibited a 21% probability of toxicity. Significant univariate linear relationship was found between RI and mean PEL quotient, suggesting that both are effective methods for ecological risk. Cr, Mn, Co, Zn, and Pb were influenced not only by nature, but also by mariculture activities; Cu was mainly derived from land runoff input and mariculture, and Ni was mainly derived from fuel used for ship combustion.

## Figures and Tables

**Table 1:** Heavy metal concentrations in surface sediments of Zhelin Bay (mg/kg dry weight).

Region		Cr	Mn	Co	Ni	Cu	Zn	Pb
This Study	Mean	81.89	770.76	16.81	62.25	96.30	162.04	72.40
	Maximum	98.88	1132.90	19.90	123.10	159.33	201.70	85.90
	Minimum	43.40	433.60	10.00	46.90	81.13	138.90	53.70
Background value [32]		28.60	343.00	8.41	14.90	19.22	59.20 [33]	40.42 [33]
Standard of marine sediment quality [31]	ClassⅠ	≤80	n.a.	n.a.	n.a.	≤35	≤150	≤60
	ClassⅡ	≤150	n.a.	n.a.	n.a.	≤100	≤350	≤130
	ClassⅢ	≤270	n.a.	n.a.	n.a.	≤200	≤600	≤250
Threshold effects level (TEL) [34]		52.3	460	n.a.	15.9	18.7	124	30.2
Probable effects level (PEL) [34]		160	1100	n.a.	42.8	108	271	112
Quanzhou Bay [29]		84.72	1295	11.93	26.77	60.81	186.7	66.98
Shantou Bay [27]		53.56	598.86	n.a.	22.95	48.52	153.28	51.63
Daya Bay [30]		65.04	837 [25]	12.7 [25]	31.2 [25]	24.58	111.65	22.64
Pear River Esutary [24,26]		106	900	14.6	36.7	45.7	176.8	57.9
Qinzhou Bay [28]		50.18	n.a.	n.a.	27.03	27.07	73.60	46.56
San Francisco Bay [23]		19	524	n.a.	33	33	60	19

n.a.: not available.

**Table 2:** Principal component analysis results (rotated component matrix and total variance interpretation).

Parameter	PC1	PC2	PC3
Co	0.961	−0.039	−0.040
Cr	0.944	−0.032	0.032
Fe	0.926	0.014	−0.018
Al	0.814	−0.433	−0.088
Pb	0.789	0.015	−0.012
Mn	0.742	0.128	0.095
Zn	0.640	0.389	0.403
Cu	−0.389	0.797	0.059
OM	0.234	0.733	−0.039
Ni	−0.056	−0.246	0.885
Median Size Eigen value Percentage of total variance (%) Cumulative percentage variance (%)	0.064 5.144 46.78 46.78	0.451 2.032 18.48 65.24	0.748 1.277 11.61 76.85

## Data Availability

The datasets used and analyzed during the current study are available from the corresponding author on reasonable request.

## Supplementary Materials

The following supporting information can be downloaded at: https://www.mdpi.com/article/10.3390/toxics11020150/s1, Figure S1: Pollution load index (PLI) values of the studied heavy metals for each sampling site of Zhelin Bay. Table S1: The geo-accumulation index (*I_geo_*) pollution classification. Table S2: Potential ecological hazard assessment indicators. Table S3: Pearson correlation matrix of heavy metals and OM in surface sediments of Zhelin Bay (*n* = 17).
