# Supplementary material for: Distribution, Multi-Index Assessment, and Sources of Heavy Metals in Surface Sediments of Zhelin Bay, a Typical Mariculture Area in Southern China
Source: Toxics. 2023 Feb 3;11(2):150. doi: 10.3390/toxics11020150 (PMC9961395; doi:10.3390/toxics11020150)
Supplement: Supplementary file 1 [file toxics-11-00150-s001.zip › toxics-2176412-supplementary.pdf]

toxics

MDPI

# Supplementary Materials: Distribution, Multi-Index Assessment, and Sources of Heavy Metals in Surface Sediments of Zhelin Bay, a Typical Mariculture Area in Southern China

**Yan-Jie Han, Rui-Ze Liang, Hai-Song Li, Yang-Guang Gu *, Shi-Jun Jiang and Xiang-Tian Man**

**Table S1.** The geo-accumulation index ($I_{geo}$) pollution classification.

| *Igeo* | Range | The Degree of Pollution |
|---|---|---|
| ≤0 | 0 | Unpolluted |
| 0<*Igeo*≤1 | 1 | From unpolluted to moderately |
| 1<*Igeo*≤2 | 2 | Moderately polluted |
| 2<*Igeo*≤3 | 3 | From moderately to strongly |
| 3<*Igeo*≤4 | 4 | Strongly polluted |
| 4<*Igeo*≤5 | 5 | From strongly to extremely |
| >5 | 6 | Extremely polluted |

**Table S2.** Potential ecological hazard assessment indicators.

| Ecological Risk | Low | Moderate | Considerable | High | Very high |
|---|---|---|---|---|---|
| $E_r^i$ | <30 | 30~50 | 50~100 | 100~150 | ≥150 |
| RI | <100 | 100~150 | 150~200 | 200~300 | ≥300 |

**Table S3.** Pearson correlation matrix of heavy metals and OM in surface sediments of Zhelin Bay ($n$ = 17).

| Metals | Al | Fe | Cr | Mn | Co | Ni | Cu | Zn | Pb | OM | Median Size |
|---|---|---|---|---|---|---|---|---|---|---|---|
| Al | 1 | | | | | | | | | | |
| Fe | 0.719** | 1 | | | | | | | | | |
| Cr | 0.723** | 0.885** | 1 | | | | | | | | |
| Mn | 0.409* | 0.644* | 0.691** | 1 | | | | | | | |
| Co | 0.852* | 0.947* | 0.887** | 0.631** | 1 | | | | | | |
| Ni | −0.070 | −0.021 | 0.007 | 0.029 | −0.097 | 1 | | | | | |
| Cu | −0.627** | −0.321 | −0.384 | −0.275 | −0.343 | −0.133 | 1 | | | | |
| Zn | 0.386 | 0.510* | 0.608** | 0.465* | 0.604** | 0.159 | 0.232 | 1 | | | |
| Pb | 0.618** | 0.627** | 0.699** | 0.498* | 0.668** | −0.059 | −0.310 | 0.508* | 1 | | |
| OM | −0.148 | 0.256 | 0.193 | 0.292 | 0.144 | −0.074 | 0.269 | 0.198 | 0.196 | 1 | |
| Median Size | −0.151 | 0.042 | 0.028 | 0.142 | 0.036 | 0.401 | 0.305 | 0.443* | 0.059 | 0.284 | 1 |

*: $p < 0.05$, Correlation is significant at the 0.05 level. **: $p < 0.01$, Correlation is significant at the 0.01 level.

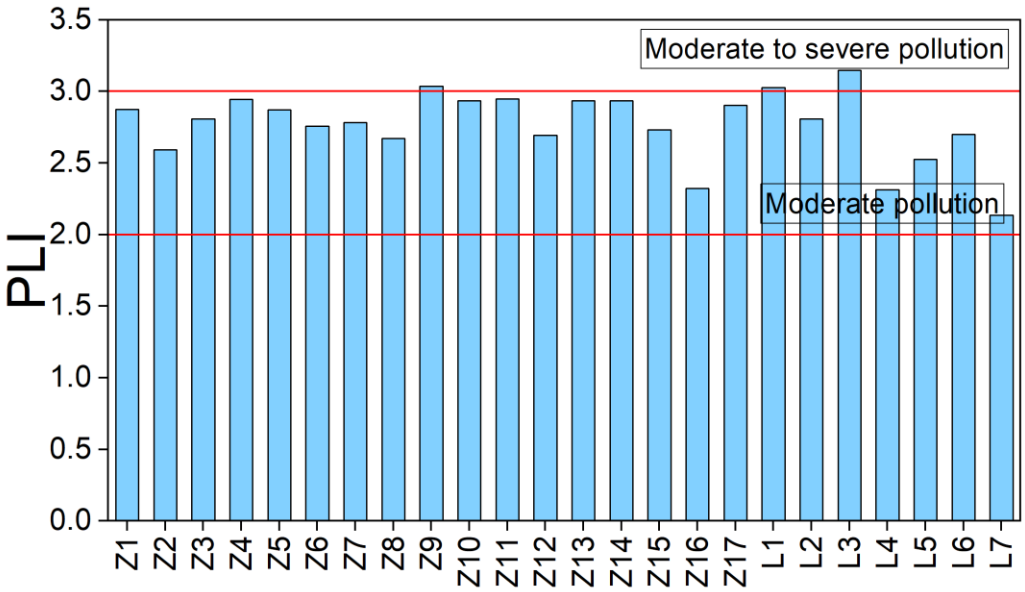


**Figure S1.** Pollution load index (PLI) values of the studied heavy metals for each sampling site of Zhelin Bay.
